# Lysophosphatidic Acid Triggers Apoptosis in HeLa Cells through the Upregulation of Tumor Necrosis Factor Receptor Superfamily Member 21

**DOI:** 10.1155/2017/2754756

**Published:** 2017-02-19

**Authors:** Yunzhou Dong, Yong Wu, Mei-Zhen Cui, Xuemin Xu

**Affiliations:** ^1^Department of Biomedical & Diagnostic Sciences, College of Veterinary Medicine, University of Tennessee, Knoxville, TN 37996, USA; ^2^Vascular Biology Program, Boston Children's Hospital, Harvard Medical School, Boston, MA 02115, USA; ^3^Division of Cancer Research and Training, Department of Internal Medicine, Charles R. Drew University of Medicine and Science, Los Angeles, CA 90059, USA; ^4^David Geffen UCLA School of Medicine and UCLA Jonson Comprehensive Cancer Center, University of California, Los Angeles, CA 90095, USA

## Abstract

Lysophosphatidic acid (LPA), a naturally occurring bioactive phospholipid, activates G protein-coupled receptors (GPCRs), leading to regulation of diverse cellular events including cell survival and apoptosis. Despite extensive studies of the signaling pathways that mediate LPA-regulated cell growth and survival, the mechanisms underlying the apoptotic effect of LPA remain largely unclear. In this study, we investigated this issue in HeLa cells. Our data demonstrate that LPA induces apoptosis in HeLa cells at pathologic concentrations with a concomitant upregulation of the expression of TNFRSF21 (tumor necrosis factor receptor superfamily member 21), also known as death receptor number 6 (DR6) involved in inflammation. Moreover, treatment of cells with LPA receptor (LPAR) antagonist abolished the DR6 upregulation by LPA. LPA-induced DR6 expression was also abrogated by pertussis toxin (PTX), an inhibitor of GPCRs, and by inhibitors of PI3K, PKC, MEK, and ERK. Intriguingly, LPA-induced DR6 expression was specifically blocked by dominant-negative form of PKC*δ* (PKC*δ*-DN). LPA-induced DR6 expression was also dramatically inhibited by knockdown of ERK or CREB. These results suggest that activation of the MEK/ERK pathway and the transcription factor CREB mediate LPA-induced DR6 expression. More interestingly, knockdown of DR6 using siRNA approach remarkably attenuated LPA-induced apoptosis. In conclusion, our results suggest that LPA-induced apoptosis in HeLa cells is mediated by the upregulation of DR6 expression.

## 1. Introduction

Lysophosphatidic acid (1- or 2-acyl-lysophosphatidic acid, LPA) is a naturally occurring bioactive phospholipid. Under normal physiological conditions, LPA is present at a low level in plasma (~100 nM) [1] and its concentration elevates at sites of tissue injury and inflammation [1, 2]. LPA is produced during phospholipid biosynthesis of cell membranes. LPA is also produced extracellularly by several cell types including activated platelets, leukocytes, epithelial cells, neuronal cells, and tumor cells [3]. Increasing evidence suggests that LPA plays a role in various inflammatory disease [4–6]. LPA regulates various developmental, physiological, and pathophysiological processes including cell motility, invasion, proliferation, survival, and production of growth factors [7, 8] through G protein-coupled receptor (GPCR). Platelet-derived LPA plays an important role in tissue regeneration and wound healing [9, 10]. LPA also functions as a chemoattractant promoting motility of various types of human cancer cells [11]. Recent studies suggest that tumor cells stimulate the production of LPA from activated platelets, which enhances both tumor growth and cytokine-mediated bone destruction [12, 13], and that specific inhibition of LPA receptors abolishes the migration of cancer cell response to malignant ascites containing LPA [14]. The increased expression of LPA receptors is associated with an increased level of invasiveness in ovarian cancer cells [15, 16]. Thus, LPA has diverse biological activities implicated in tumor initiation and progression, including increasing cell survival, angiogenesis, invasion, and metastasis. Paradoxically, LPA also induces apoptosis in certain cells, such as neuronal cells [17] and epithelial cells from different tissues [18–20], myeloid progenitor cells, hippocampal neurons, and PC12 cells [3]. However, the mechanism by which LPA induces apoptosis remains unclear.

Death receptor 6 (DR6), also known as TNFRSF21, is a relatively new member of the tumor necrosis factor (TNF) receptor family possessing a cytoplasmic death domain. Previous studies indicate that ectopic expression of DR6 in mammalian cells induces apoptosis [21]. Our recent study suggests that DR6 induces apoptosis through a mitochondria-dependent pathway [22]. In the current study, to determine the mechanism by which LPA induces apoptosis, we surprisingly found that LPA induced apoptosis via upregulating expression of the death receptor DR6 in HeLa cells. In addition, our results strongly suggest that PI3K/PKC/MEK/ERK pathway and activation of the transcription factor CREB are responsible for LPA-induced upregulation of DR6 expression and apoptosis.

## 2. Materials and Methods

### 2.1. Reagents, Chemicals, and Antibodies

Anti-DR6 antibody was purchased from Santa Cruz Biotechnology (Santa Cruz, CA). Phosphor antibodies for PKC*α*/*β*, PKC*ε*, PKC*ζ*/*λ*, PKC-delta, MEK, ERK1/2, p90RSK, CREB, cleaved caspases 9, 7, 3, and PARP were purchased from Cell Signaling Technology (Danvers, MA). Antibody against LPAR1 was purchased from Cayman Chemical (Ann Arbor, MI); antibodies for LPAR2 and LPAR3 were from LifeSpan BioScience (Seattle, WA). LPA (category number 857130, water-soluble) and LPS were from Avanti Polar Lipids (Alabaster, AL). TNF-*α*, angiotensin II, PMA, insulin, thrombin, NGF-*β*, Ro 31-8220 (PKC inhibitor), and SB203580 (p38 inhibitor) were bought from Sigma-Aldrich (St. Louis, MO). Pertussis toxin (PTX), UO126, and GF10293X were from Biomol International (Plymouth Meeting, PA). Wortmannin (PI3K inhibitor) and Ki16425 (LPA Receptor Antagonist) were from Cayman Chemical (Boston, MA). ERK and CREB siRNA were from Cell Signaling Technology. DR6 siRNA was bought from Santa Cruz Biotechnology (CA). JNK inhibitor was purchased from Calbiochem (Billerica, MA). MTT assay kit for cell viability was bought from R&D System (Minneapolis, MN). TUNEL assay kit was purchased from Roche Applied Science USA. Adenoviral vector construction kit was from Qbiogene. Cloning tools including restriction enzymes, T4 DNA ligase, and modifying enzymes were bought from New England Biolabs or Promega. All other chemicals were purchased from Sigma-Aldrich or other brand companies.

### 2.2. Cell Culture and Treatment

HeLa cells were cultured in DMEM with 10% DMEM and antibiotics (Penn/Strep). Cells were starved for 16 hours before LPA treatment or as indicated in each experiment. Inhibitors were usually pretreated for 30 min followed by LPA treatment except the PTX pretreatment for 15 hours.

### 2.3. Northern Blot

Northern blot was performed as described previously [23]. Total RNA was extracted using TRIzol reagent (Invitrogen) and subjected to formaldehyde-agarose gel electrophoresis. RNA was transferred to nylon membranes (Amersham Biosciences), UV-crosslinked and hybridized with dCTP-labeled 444-bp fragment (see Supplemental Figure 2, in Supplementary Material available online at https://doi.org/10.1155/2017/2754756) of human DR6 cDNA. 18S and 28S rRNA were used as internal controls. Intensities of hybridized bands were quantified using Image J software.

### 2.4. Western Blot

Western blot was conducted as previously described [24]. In brief, cells were washed with cold PBS one time and immediately lysed in lysis buffer containing 50 mM Tris-HCl, pH 6.8, 8 M urea, 5%  *β*-mercaptoethanol, 2% SDS, 0.2 mM NaF, 0.1 mM Na_2_VO_3_, and protease inhibitors (Roche). Protein concentration was determined by BCA kit (Fisher Scientific) for equal amount of total protein loading. Proteins were separated in Tris-glycine SDS-PAGE gel and transferred to PVDF membrane (Fisher Scientific), followed by probing with different antibodies and visualized by ECL reagent.

### 2.5. PCR, Reverse Transcriptional PCR, and qPCR

PCR primers were designed for human LPAR1, LPAR2, and LPAR3 for regular PCR. DR6 primers were designed for the amplification of human DR6 message RNA for Reverse Transcriptional (RT-) PCR. Primers have also been designed for the DR6 for Quantitative PCR (qPCR) analysis. All primers for targeting gene amplification and the relative controls are supplemented in Supplemental Table 1. Regular and reverse transcriptional PCR were performed with a Bio-Rad thermocycler using the following program: 95°C 2 min, followed by 32 cycles of 95°C 15 s, 55°C 30 s, and 72°C 45 s followed by extension at 72°C for 7 min. For reverse transcriptional PCR, the cycle number was controlled between 15 and 20 cycles and the PCR products were subjected to 1% agarose gel electrophoresis. Quantitative PCR was performed as previously reported [25]. In brief, the qPCR mixture was heated to 95°C for 3 min and then subjected to 40 cycles at 95°C for 30 s, 57°C for 30 s, and 72°C for 1 min by using the MyiQ™ System (Bio-Rad). The cycle threshold (Ct) value was determined for each sample. All Ct values were normalized to the internal control gene *β*-actin. The relative expression of DR6 mRNA, as determined by Ct value, was calculated using the equation 2^−Δct^.

### 2.6. Adenoviral Infection

Adenovirus harboring different dominant-negative (DN) PKC isoforms and DR6 was titrated and added to cell culture at the 50x MOI (multiplicity of infection) for 24 hrs.

### 2.7. Downregulation of the Expression of DR6, ERK, and CREB by siRNA

Downregulating the expression of ERK and CREB by siRNA technology was performed following the protocol from the supplier. Two rounds of siRNA treatment were conducted for a better knockdown efficiency.

### 2.8. MTT and TUNEL Assay

Cells were treated with LPA in different concentration and time points as indicated and MTT assay was performed according to the recommendation by supplier. For terminal deoxynucleotidyl transferase- (TdT-) mediated dUTP nick end labeling (TUNEL) assay, cells were treated with 25 *μ*M LPA for 24 hrs, fixed, and stained. Images were captured using Olympus fluorescent microscopy equipped with a digital camera.

### 2.9. Statistical Analysis

All experiments were repeated for at least three times. Image bands from Western blot, Northern blot, and PCR/qPCRs were quantified using Image J software (NIH website). Data was presented as mean ± SD. Statistical analysis was performed using Prism software for comparison by either Student's *t*-test (two groups) or one-way ANOVA (≥three groups). A *P* value of less than 0.05 was considered a significant difference.

## 3. Results

### 3.1. LPA Induces Apoptosis and DR6 Expression in Cultured HeLa Cells

**Figure 1:**
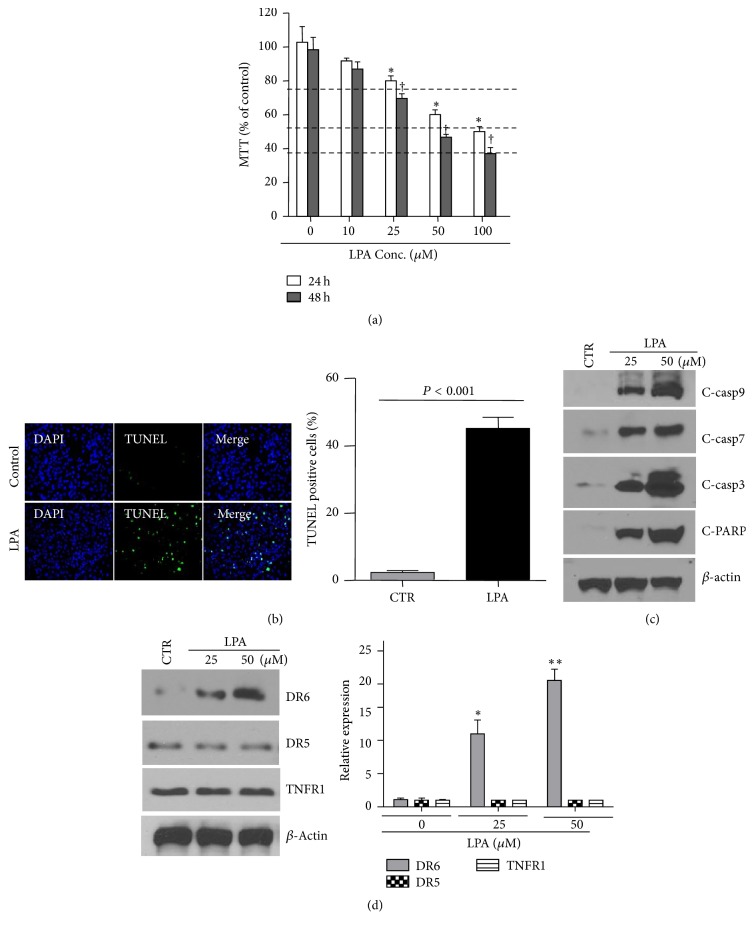
LPA induces apoptosis accompanied by upregulation of DR6 expression. (a) LPA treatment reduced cell viability in a concentration-dependent manner as determined by MTT assay. 10–25 *μ*M LPA-induced significant cell viability reduction compared to control. *n* = 4, ^*∗*^*P* < 0.05 versus control; ^†^high concentration of LPA at 50–100 *μ*M caused more cell viability reduction. *n* = 4. (b) LPA-triggered apoptosis was determined by TUNEL staining. HeLa cells were treated by 25 *μ*M LPA for 24 hrs. *n* = 3. The bar graphs on the right panel represent quantification of TUNEL assay, *n* = 3, *P* < 0.001 versus control. (c and d) HeLa cells were exposed to different concentration of LPA for 18 hours. Activation of caspase-9, caspase-7, and caspase-3 and the cleavage of PARP (c), and expression levels of DR6, DR5, and TNFR (d) were determined by Western blot. The blot is a representative of 4 blots from 4 independent experiments (*n* = 4). The bar graphs on the right panel are densitometry analyses of DR6, DR5, and TNFR1 protein expression. ^*∗*^*P* < 0.05, ^*∗∗*^*P* < 0.001 versus control.

To test whether LPA can induce apoptosis, HeLa cells were treated with various concentrations of LPA for up to 48 hrs. LPA-induced apoptosis in HeLa cells was determined by MTT and TUNEl assay. As shown in Figures 1(a) and 1(b), the reduction of cell viability determined by MTT assay and the increase in the number of TUNEL-positive cells indicate that the apoptotic effect was apparently dose-dependent with the lowest levels at 10 *µ*M of LPA treatment. The proapoptotic effect of LPA in HeLa cells was confirmed with apoptotic signaling protein activation. As shown in Figure 1(c), LPA treatment (25 and 50 *μ*M) significantly increased caspase-9, caspase-7, and caspase-3 activation and PARP cleavage. Interestingly, as shown in Figure 1(d), the dose-dependent proapoptotic effect of LPA was accompanied by gradual augmentation of expression of DR6, a recently identified death receptor member of TNF superfamily. Treatment with LPA did not change the levels of DR5 and TNFR1, the other two members of the same TNF superfamily.

### 3.2. LPA Increases DR6 mRNA and Protein Expression in Both Dose- and Time-Dependent Manner

**Figure 2:**
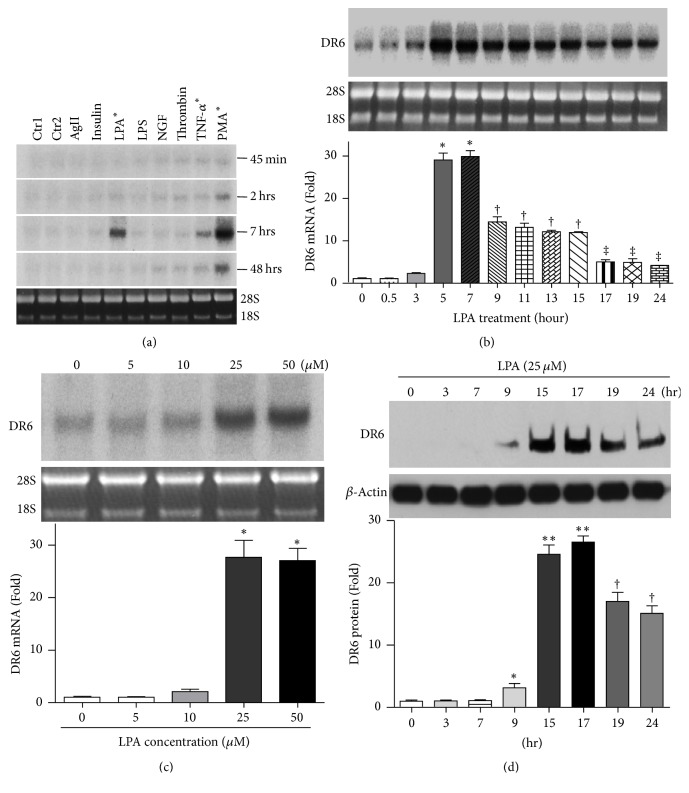
LPA upregulates DR6 expression in does- and time-dependent manner. (a) HeLa cells were exposed to various stimuli and the expression of DR6 was measured by Northern blot at different time points as indicated. Control 1: untreatment; Control 2: vehicle (DMSO) treatment. ^*∗*^*P* < 0.001 versus control. (b) HeLa cells were treated with LPA (25 *μ*M) for different times as indicated. Expression of DR6 was measured by Northern blot. *n* = 3. ^*∗*^*P* < 0.001 versus control; ^†^*P* < 0.05 versus 5–7 hr time point, ^‡^*P* < 0.05 versus 9–15 hr time points. (c) HeLa cells were treated with various concentrations of LPA for 16 hrs. DR6 mRNA expression was measured by Northern blot. *n* = 3, ^*∗*^*P* < 0.001 versus control. (d) HeLa cells were treated with LPA 25 *μ*M for the times indicated. The cells were then lysed, and 50 *μ*g of the cell lysates was analyzed by SDS-PAGE followed by Western blotting with anti-DR6 antibody and then were visualized by the enzyme-linked chemiluminescence system. The bar graphs below are densitometry analyses of DR6 protein expression. Data presented are mean ± SD from 3 independent experiments, with untreated controls set as 1. ^*∗*^*P* < 0.05, ^*∗∗*^*P* < 0.001 versus control; ^†^*P* < 0.05 versus 15–17 time points.

Next, we compared the effects of different proapoptotic factors and growth factors on DR6 expression. HeLa cells were treated with various stimuli including 0.1 *μ*M angiotensin II (AgII), 20 ng/mL insulin, 25 *μ*M LPA, 1 *μ*g/mL LPS, 50 ng/mL NGF-*β*, 0.1 unit/mL thrombin, 50 ng/mL TNF-*α*, and 100 ng/mL phorbol 12-myristate 13-acetate (PMA). The expression of DR6 mRNA was measured by Northern blot at different time points as indicated. As shown in Figure 2(a), it was noted that only TNF-*α*, PMA, and LPA significantly upregulated DR6 expression at 7 hrs. TNF-*α* has been known to induce DR6 in several cancer cell lines [26]. PMA has also been reported to upregulate DR6 expression during T-cell activation [27]. As shown in Figure 2(b), DR6 mRNA expression in HeLa cells treated with 25 *μ*M LPA increased transiently, with a peak level at 5–7 hr. As shown in Figure 2(c), LPA upregulated DR6 mRNA expression in a dose-dependent manner and reached a plateau at 25–50 *μ*M. This result is further confirmed by RT-PCR (Supplemental Figure 1). Next, we sought to determine whether LPA regulates DR6 protein expression following a similar time course. As shown in Figure 2(d), 25 *μ*M LPA treatment transiently increased DR6 protein expression in HeLa cells as early as 9 hr, with a peak level at 15–17 hr.

### 3.3. LPA Receptors 1 and 3 Mediate LPA-Induced DR6 Upregulation

**Figure 3:**
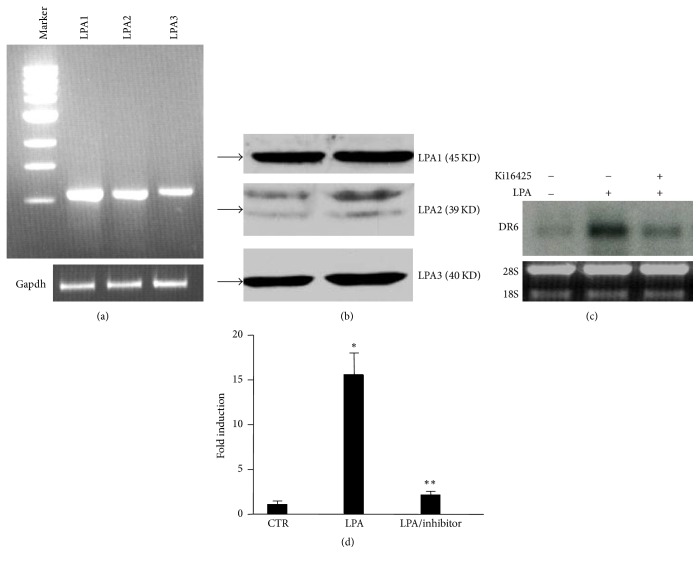
LPA-induced DR6 upregulation is mediated by LPA receptors 1/3 (LPAR1/3). (a and b), LPA1, LPA2, and LPA3 mRNA and protein are all expressed in HeLa cells, as indicated by PCR and Western blot, respectively. *n* = 3. (c) LPA1/3 antagonist Ki16425 (3 *μ*M) attenuated the DR6 upregulation induced by LPA. *n* = 4. (d) The bar graphs are statistical analysis of DR6 expression. Data presented are mean ± SD from 4 independent experiments, with untreated controls set as 1. ^*∗*^*P* < 0.001 versus control; ^**^P < 0.001 versus LPA.

Our data revealed that LPA receptors 1–3 (LPAR1–3) were expressed in HeLa cells (Figures 3(a) and 3(b)). To determine the role of LPAR in LPA-stimulated DR6 upregulation, we treated the cells with Ki16425 (3 *μ*M), an LPA1/3 antagonist prior addition of LPA. As shown in Figures 3(c) and 3(d), pretreatment of cells with Ki16425 strongly inhibited LPA-induced DR6 expression, suggesting that LPA-induced DR6 upregulation was mediated by LPA receptors 1 and 3.

### 3.4. PI3K, PKC, and MEK Pathways Are Responsible for LPA-Stimulated DR6 Expression

**Figure 4:**
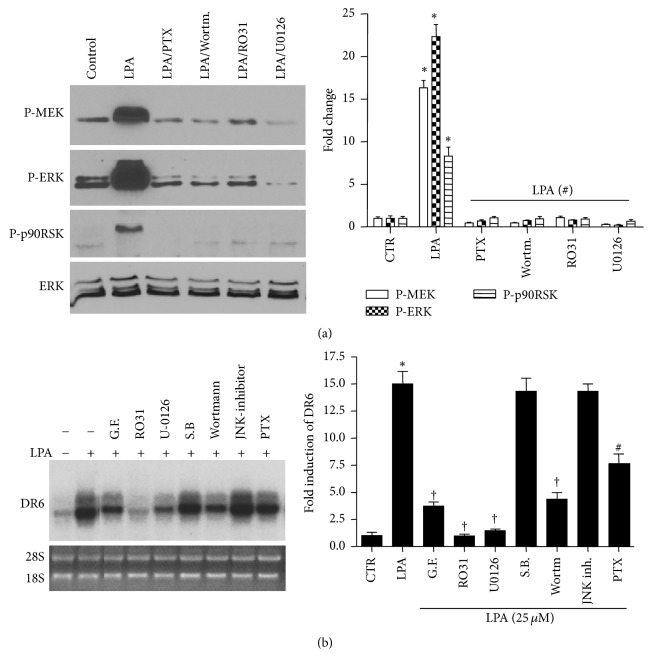
PI3K, PKC, and MEK pathways are involved in LPA-induced DR6 expression. (a) HeLa cells were treated with LPA (25 *μ*M) in the presence or absence of the pathway inhibitors as indicated. The phosphorylation of MEK, ERK, and p90RSK was analyzed by Western blotting. The blot is a representative of 3 independent experiments. The bar graphs on the right panel are densitometry analyses of MEK, ERK, and p90RSK phosphorylation levels. ^*∗*^*P* < 0.001 versus control; ^#^*P* < 0.001 versus LPA-treated group. (b) HeLa cells were treated with LPA in the presence or absence of the pathway inhibitors as indicated and the expression of DR6 was measured by Northern blot. The bar graphs on the right panel are statistical analysis of DR6 expression. Data presented are mean ± SD from 3 independent experiments, with untreated controls set as 1. ^*∗*^*P* < 0.001 versus control; ^†^*P* < 0.001 versus LPA alone; ^#^*P* < 0.001 versus LPA-treated group. PTX, pertussis toxin (100 ng/mL); GF10293X, PKC inhibitor (5 *μ*M); wortmannin, PI3K inhibitor, (100 nM); Ro 31-8220, PKC inhibitor (5 *μ*M); U-0126, MEK inhibitor (10 *μ*M); SB203580, p38 inhibitor (2.5 *μ*M) and JNK inhibitor (10 *μ*M).

As shown in Figure 4(a), treatment with LPA significantly induced MEK, ERK, and p90RSK phosphorylation. To determine the mechanism underlying LPA-induced DR6 expression, we first examined the effect of pertussis toxin (PTX), which inactivates the LPA receptor-coupled Gi/o type G protein [28], as shown in Figure 4(a); treatment with PTX inhibited LPA-induced phosphorylation of MEK, ERK, and p90RSK. LPA-induced phosphorylation of MEK, ERK, and p90RSK was also inhibited by wortmannin, a PI3K inhibitor, Ro 31-8220, a PKC inhibitor, and U-0126, a MEK inhibitor (Figure 4(a)). Next, we examined the roles of these kinases in LPA-induced DR6 expression. As shown in Figure 4(b), LPA-induced increase in the level of DR6 mRNA was strongly inhibited by Ro 31-8220, a cell-permeable inhibitor of PKC isoforms PKC*α*, PKC*β*, PKC*γ*, and PKC*ε*. Another PKC inhibitor GF 109203X also significantly inhibited LPA-induced DR6 mRNA expression, but not as strongly as Ro 31-8220. In addition to PKC inhibitors, the MEK inhibitor U-0126 and PI3K inhibitor wortmannin also significantly inhibited LPA-induced DR6 mRNA expression. The G protein inhibitor, PTX, inhibited LPA-induced DR6 mRNA expression too, but to a lesser extent. On the other hand, inhibition of p38 and JNK activity with SB203580 and JNK inhibitor had no effect on LPA-induced increase in DR6 mRNA expression. Together, our results suggested that PI3K/PKC/MEK pathway mediates LPA-induced DR6 expression.

### 3.5. PKC-Delta Specifically Mediates LPA-Induced DR6 Expression

**Figure 5:**
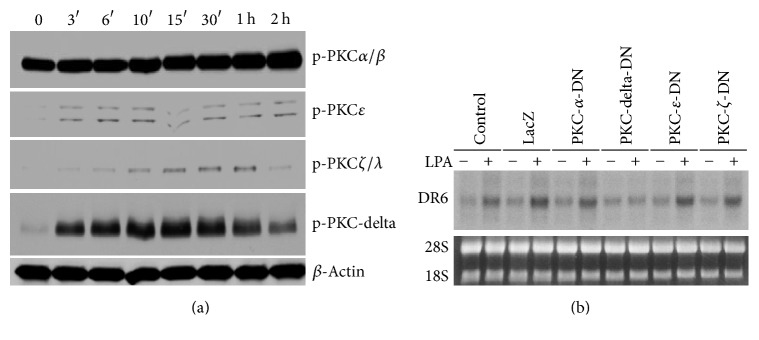
LPA-induced DR6 upregulation is specifically mediated by PKC*δ* isoform. (a) A time course of LPA-induced activation of PKC kinases. The blots were incubated in sequence with antibodies against several major PKC isoforms such as PKC*α*/*β*, PKC*ε*, PKC*ζ*/*λ*, and PKC*δ*. All isozymes stained as a dominant band of the expected molecular weight. *n* = 3. (b) HeLa cells were infected with adenoviral PKC*α*-DN, PKC*δ*-DN, PKC*ε*-DN, and PKC*ζ*-DN for up to 24 hrs. Cells were then starved in DMEM serum-free overnight and stimulated with 25 *μ*M LPA (+) or vehicle (−) for 8 hrs, followed by Northern blotting for DR6 mRNA expression. *n* = 3.

Data presented in Figure 4 strongly suggest that activation of PKC is functionally involved in LPA-induced DR6 expression. To determine the specific isoform of PKC that contributes to LPA-induced DR6 expression, we first determined the LPA-induced activation of major PKC isoforms, PKC*α*/*β*, PKC*ε*, PKC*ζ*/*λ*, and PKC*δ*. HeLa cells were treated with 25 *μ*M LPA for different time as indicated and the activation of the PKC isoforms was detected by Western blot analysis. As shown in Figure 5(a), upon treatment with LPA, all PKCs examined were activated to various extents. Among them, the high level of basal phosphorylation of PKC*α*/*β* was not significantly affected by LPA treatment. Similarly, the phosphorylation levels of PKC*ε* and PKC*ζ*/*λ* were also slightly increased by LPA treatment during the time course. On the other hand, PKC*δ* was remarkably activated upon treatment with LPA as determined by the rapid and robust increase in the phosphorylation level of it. Next, we determined the effect of expression of dominant-negative forms of these PKC isoforms on the LPA-induced DR6 expression. As shown in Figure 5(b), expression of dominant-negative forms of PKC*α*, PKC*ε*, and PKC*ζ* had no effect on LPA-induced DR6 expression. However, expression of dominant-negative form of PKC*δ* almost completely blocked LPA-induced DR6 mRNA expression. Together, our data strongly suggest that PKC*δ* is the major PKC isoform that is functionally involved in signaling pathway that mediates LPA-induced DR6 expression. These data demonstrated for the first time PKC isoform-specific transcriptional regulation of LPA-induced DR6 expression.

### 3.6. Phosphorylated ERK and CREB Participate in LPA-Stimulated DR6 Expression

**Figure 6:**
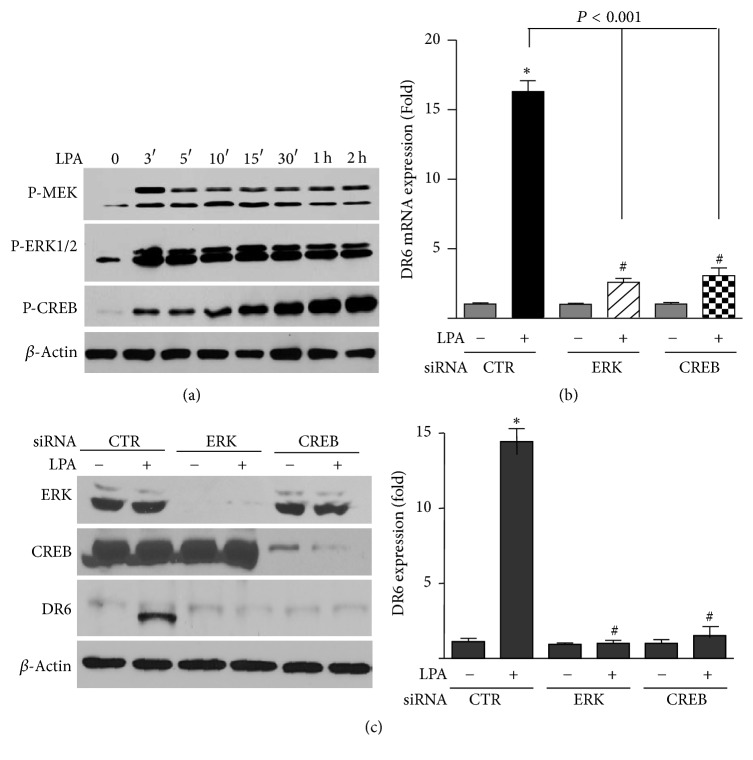
ERK and CREB pathway regulates LPA-induced DR6 expression. (a) HeLa cells were treated with LPA (25 *μ*M) at various concentrations as indicated. The phosphorylation of MEK, ERK, and CREB was analyzed by Western blotting. The blot is a representative of 3 independent experiments (*n* = 3). (b and c) HeLa cells were treated with LPA in the presence of control siRNA and ERK- or CREB-specific siRNAs. The expressions of DR6 mRNA (b) and protein (c) were measured by q-PCR and Western analysis, respectively. The bar graphs on the right panel of (c) are statistical analysis of DR6 expression. Data presented are mean ± SD from 3 independent experiments, with nontreated controls set as 1. ^*∗*^*P* < 0.001 versus control; ^#^*P* < 0.001 versus LPA/control siRNA.

Data presented in Figure 4 showed that LPA induced the activation of both ERK and p90RSK. Previous study has reported that LPA can induce p90RSK activation through ERK [29]. The serine/threonine kinase p90Rsk is known to directly phosphorylate the transcriptional factor CREB (cAMP response element-binding protein) [30, 31]. Thus, we thought to determine whether LPA-induced activation of MEK-ERK-90RSK pathway leads to the activation of CREB and whether CREB is involved in LPA-induced expression of DR6. As shown in Figure 6(a), LPA induced activation of MEK and ERK1/2 at a very early time point. Moreover, LPA also strongly induced the activation of the transcription factor CREB in a time-dependent manner. To determine the role of these molecules in LPA-regulated DR6 expression, we employed a siRNA approach. As shown in Figure 6(b), knockdown of ERK and CREB strongly blocked LPA-induced DR6 mRNA expression. As shown in Figure 6(c), Western blot analysis confirmed the efficient knockdown of the expression of ERK and CREB. As expected, the protein level of DR6 was dramatically reduced in ERK- and CREB-knockdown cells. These observations suggest that LPA induces DR6 expression through activation of the PKC*δ*-ERK signaling pathway and the subsequent activation of CREB.

### 3.7. Downregulation of DR6 by siRNA Attenuates LPA-Induced Apoptosis

**Figure 7:**
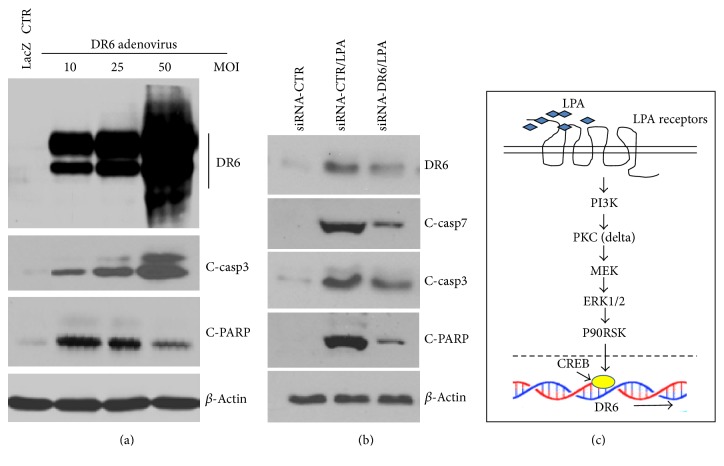
DR6 induction is responsible for LPA-induced apoptosis and sketch of mechanism. (a) Overexpression of DR6 induced apoptosis. HeLa cells were transiently infected with adenoviruses encoding constitutively active DR6 with different multiplicity of infection (MOI); or a control adenovirus carrying the LacZ gene (LacZ). After 24 hr infection, cells were harvested, and the lysates were separated by SDS-PAGE, followed by Western blot analysis for DR6 expression, caspase activation, and PARP cleavage. This membrane was also reprobed with anti-*β*-actin antibody to indicate relative loading of samples (bottom panel). The blot is a representative of 3 independent experiments (*n* = 3). (b) Downregulation of DR6 expression by siRNA attenuates LPA-triggered apoptosis. HeLa cells were treated with LPA in the presence of control siRNA or DR6 siRNA. The expressions of DR6 and activation of caspase-7, caspase-3, and PARP cleavage were measured by Western analysis. *n* = 4. (c) The proposed molecular mechanism of LPA-induced DR6 expression in HeLa cells. LPA ligand binds to its receptors LPAR1 or LPAR3 to initiate the cell signaling by activation PI3K, followed by the activation of PKC *δ* and MEK, which phosphorylate ERK1/2. ERK then activates CREB probably mediated by activation of P90-RSK.

Next, we further substantiated the role of DR6 in LPA-induced apoptosis. First, to determine the apoptotic activity of DR6, HeLa cells were infected with adenoviruses expressing constitutively active DR6. As shown in Figure 7(a), infection of DR6-expressing adenovirus resulted in a dose-dependent apoptosis as determined by the cleavage of PARP, an indicator of apoptosis [32], and the activation of caspase-3. As a control, no apoptotic sign was detected in cells infected with virus expressing LacZ protein. Taken together, these results clearly indicate that overexpression of DR6 induces apoptosis in HeLa cells. Next, to determine whether LPA-upregulated DR6 expression accounts for the apoptosis induced by LPA, we determined the effect of knockdown of DR6 on LPA-induced apoptosis. As shown in Figure 7(b), as expected, treatment with DR6-specific siRNA strongly inhibited DR6 expression as determined by the reduction in the DR6 protein level. Interestingly, DR6-siRNA treatment also strongly inhibited LPA-induced apoptosis as determined by the reduction of caspase activation and PARP cleavage. These results strongly suggest that LPA-induced apoptosis is mediated by induction of DR6 expression.

## 4. Discussion

LPA, a simple bioactive phospholipid, is present in biological fluids such as plasma and cerebrospinal fluid (CSF). LPA concentrations in blood can range from 0.1 *μ*M in plasma and up to 10 *μ*M in serum [33]. In this study, most of the experiments were carried out using 25 *μ*M LPA, which is in the range of pathological concentrations found in atherosclerotic lesions in vivo [34] and acute myocardial infarction [35]. This concentration range has been used to determine the apoptotic activity of LPA in other cell types, such as epithelial cells [18] and neuronal cells [17].

LPA, through its G protein-coupled receptors, regulates diverse cellular processes, including cell survival and apoptosis in many different cell types [28]. The function of LPA as a cell survival factor has been well studied; however, the mechanism by which LPA induces apoptosis remains elusive. In the current study, using pharmaceutical inhibitors, dominant-negative mutations, and siRNA approach, we explored the mechanism(s) underlying LPA-induced apoptosis. To this end, our results revealed several interesting findings. First, LPA induced apoptosis at pathologic concentrations in HeLa cells. Second, treatment with LPA resulted in remarkable upregulated expression of DR6. Third, knockdown of DR6 strongly blocked LPA-induced apoptosis. Fourth, LPA receptors 1 and 3 antagonist inhibited LPA-induced DR6 expression. Finally, inhibition of various kinases, PI3K/PKC/MEK/ERK, and their regulated transcription factor CREB strongly inhibited LPA-induced DR6 expression. Together, these results strongly suggest that LPA-induced apoptosis is mediated by upregulation of DR6 expression via activation of PI3K/PKC/MEK/ERK pathways leading to the activation of the transcription factor CREB, which controls the expression of DR6.

In this study, the first and novel finding is the identification of DR6 as an effector that mediates LPA-induced apoptosis. DR6 is a relatively less characterized member of the TNF death receptor family of protein. To date, DR6 still remains as an orphan receptor and no specific cognate ligand for it has yet been identified. Most of our current knowledge about the cellular and physiological role and biological function of DR6 comes from studying the effect of DR6 gene ablation in mice or ectopic expression of DR6 in cultured cells [36]. Recent studies reported that DR6 expression is upregulated in neurodegenerative disease such as Alzheimer's disease (AD) and amyotrophic lateral sclerosis (ALS) and the upregulated expression of DR6 may contribute to the pathogenesis of these neurodegenerative disorders [37, 38]. Interestingly, LPA has also been implicated in apoptotic neurodegeneration [17, 39–41]; however, the mechanism by which LPA induces apoptotic cell death remains elusive. In this study, our data demonstrated for the first time that LPA upregulated DR6 expression and that knockdown of DR6 strongly blocked LPA-induced apoptosis, strongly suggesting that LPA induces apoptosis via upregulating DR6 expression. Thus, our finding that LPA-induced apoptosis was mediated by upregulation of DR6 expression suggests a possibility that LPA may contribute to neurodegeneration through elevating DR6 protein level.

The second interesting discovery of this study is the finding that LPA-induced DR6 expression is mediated by activation of transcription factor CREB. Studies have reported that exogenous expression of DR6 could mimic ligand activation and triggered ligand-independent downstream apoptotic signaling cascades [21, 22]. However, although overexpression of DR6 causes apoptotic cell death, it has been found that the expression level of DR6 is elevated in several tumors in humans and the elevated expression of DR6 is regulated through activation of NF-kB [26]. Furthermore, these authors have also demonstrated that expression of DR6 expression could be induced by tumor necrosis factor-*α* (TNF-*α*) treatment and TNF-*α*-induced DR6 expression is also mediated by activation of NF-kB [26]. In contrast to this finding, it was recently reported that activation of NF-kB inhibited (E)-2,4-Bis(p-hydroxyphenyl)-2-butenal-induced DR6 expression in colon cancer cells [42]. These controversial observations might have resulted from the use of different experimental systems, such as different cell lines and different stimuli, in these studies. Interestingly, in the current study, our data demonstrated that LPA-induced DR6 expression is mediated by the activation of CREB. Our finding further supports the assumption that different stimuli activate different pathways and lead to activation of different transcriptional factors that govern the expression of DR6.

LPA is well known as a survival factor. However, it has also been reported that LPA can promote apoptosis in some cell types, including epithelial cells [18], the differentiated neuron model PC12 cells [39], and hippocampal neurons [40]. Regarding the seemingly contradictory effects of LPA on promoting cell survival and apoptosis, one possibility is that the concentration of LPA used in inducing apoptosis is higher than that used in cell survival. For example, the concentration of LPA in promoting cell survival is in the 10 *μ*M range [43, 44]. On the other hand, the concentrations of LPA used to induce apoptosis are mostly higher than 20 *μ*M [17, 18, 39, 40]. Other possibilities are that different cell types express different isoforms of LPA receptors and have unique gene expression and regulation patterns that are variably altered by LPA signals, as reviewed previously [3].

In summary, we have identified a signaling pathway implicated in LPA-induced apoptosis. This pathway is mediated by LPA receptor and its downstream PI3K/PKC/MEK/ERK signaling cascades. The most interesting and novel finding of current study is the identification of DR6 as a key factor that mediates LPA-induced apoptosis. The mechanism by which LPA functions as a survival factor has been extensively studied. However, the mechanism by which LPA regulates cell apoptosis remains largely unknown. Thus, our finding that LPA can stimulate DR6 expression leading to apoptotic cell death would contribute to better understanding of the mechanism by which LPA regulates apoptosis and identification of new therapeutic targets.

## Supplementary Material

Supplementary Material For the primers of DR6 gene expression in real-time PCR analysis (Supplementary table 1) , the results obtained from the real-time PCR amplification for DR6 expression stimulated by LPA treatment (Supplementary Figure 1), and the physical map of the transfer vector adenovirus overexpression system (Supplementary Figure 2) for DR6, readers can refer to the online supplement.
