# Supplementary material for: Lysophosphatidic Acid Triggers Apoptosis in HeLa Cells through the Upregulation of Tumor Necrosis Factor Receptor Superfamily Member 21
Source: Mediators Inflamm. 2017 Feb 19;2017:2754756. doi: 10.1155/2017/2754756 (PMC5350427; doi:10.1155/2017/2754756)
Supplement: Supplementary file 1 — Supplementary Material For the primers of DR6 gene expression in real-time PCR analysis (Supplementary table 1) , the results obtained from the real-time PCR amplification for DR6 expression stimulated by LPA treatment (Supplementary Figure 1), and the physical map of the transfer vector adenovirus overexpression system (Supplementary Figure 2) for DR6, readers can refer to the online supplement. [file 2754756.f1.pdf]

Supplementary Table 1 Primers for PCR, reverse transcriptional PCR and qPCR

Human Gene	Forward 5'-->3'	Reverse 5'-->3'	Expected size (bp)	Position in cDNAs	Type of PCR
**LPAR1**	CACAGCCATGAATGAACCAC	TACAGTTCCAGCCCACACTG	518	48-565	Regular PCR
**LPAR2**	TTTCACTTGAGGGCTGGTTC	GACATTGCAGGACTCACAGC	524	296-819	Regular PCR
**LPAR3**	GGCACATGTCAATCATGAGG	GCCATACATGTCCTCGTCCT	527	380-906	Regular PCR
**DR6 (1)**	CTTAGCACCACCACAGCTCA	ACAGCAGGTCAGGAAGATGG	1861	106-1966	Reverse transcription (RT-) PCR
**DR6 (2)**	TCCCTGACAACACAAGCTCA	CTCACTGGCATTGCAAAGAA	503	824-1326	Reverse transcription (RT-) PCR
**DR6 (3)**	CAGCTCAGCCAGAACAGAAG	GGCTTGTGTTGGTACAATGC	135	119-234	qPCR
**Gapdh**	AAGGTGAAGGTCGGAGTCAA	TGTGGTCATGAGTCCTTCCA	525	7-531	Regular PCR and RT-PCR control
**β-actin**	TGGACTTCGAGCAAGAGATG	GAAGGAAGGCTGGAAGAGTG	137	662-779	qPCR control

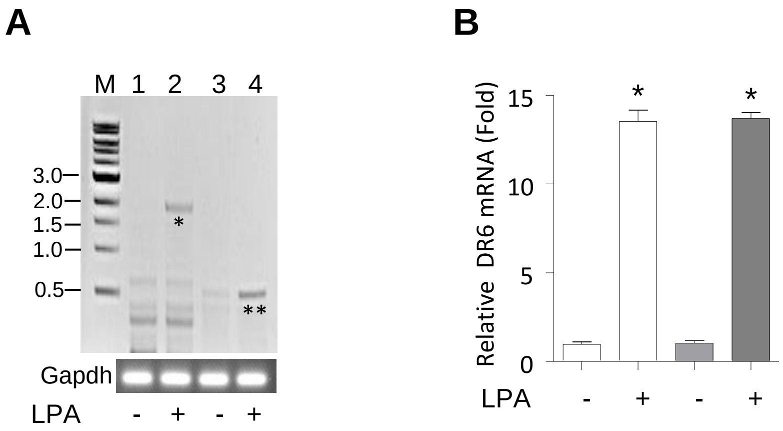

Supplementary Figure 1 LPA upregulates DR6 expression is confirmed by reverse transcriptional (RT)-PCR. (A). lane 1 & 3, control, lane 2 & 4, cells were treated by 25µM LPA treatment for 8 hours. RNA was extracted by Trizol. (B) Quantification for figure (A). n=3, * LPA treatment *vs.* control, P<0.0001.

Dong et al.

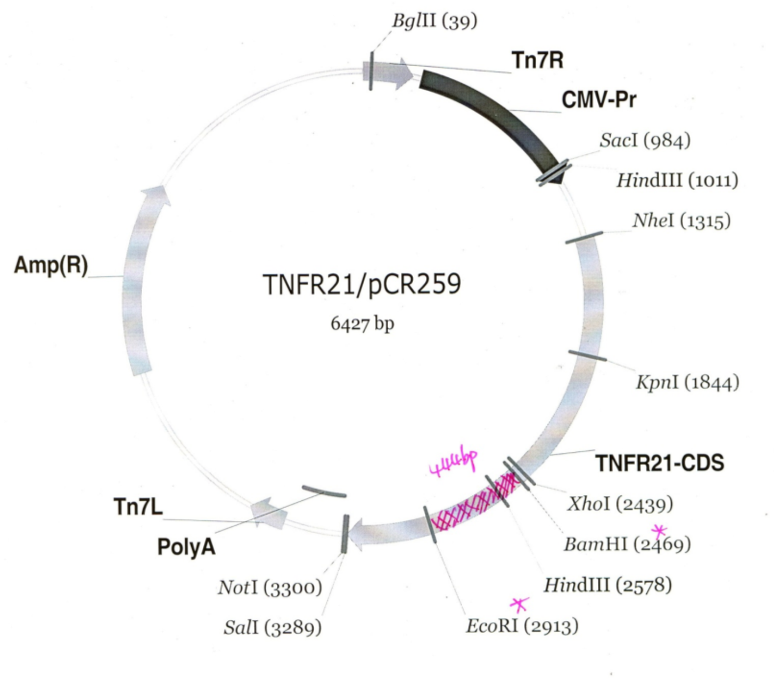

Supplementary Figure 2 DR6 (TNFR21) expression vector. DR6 was inserted into adenovirus expression system (showing DR6 cloned in transfer vector). DR6 is cloned into adenoviral transfer vector pCR259 (Q-biogene), generating TNFR21/pCR259 plasmid, which can be amplified in *Ecoli.* Figure shows the probe used in Northern blot released by EcoRI/BamHI digest (444bp length).

Dong et al.
